# Supplementary figures and images for: Male-biased aganglionic megacolon in the TashT mouse model of Hirschsprung disease involves upregulation of p53 protein activity and Ddx3y gene expression
Source: PLoS Genet. 2020 Sep 8;16(9):e1009008. doi: 10.1371/journal.pgen.1009008 (PMC7500598; doi:10.1371/journal.pgen.1009008)

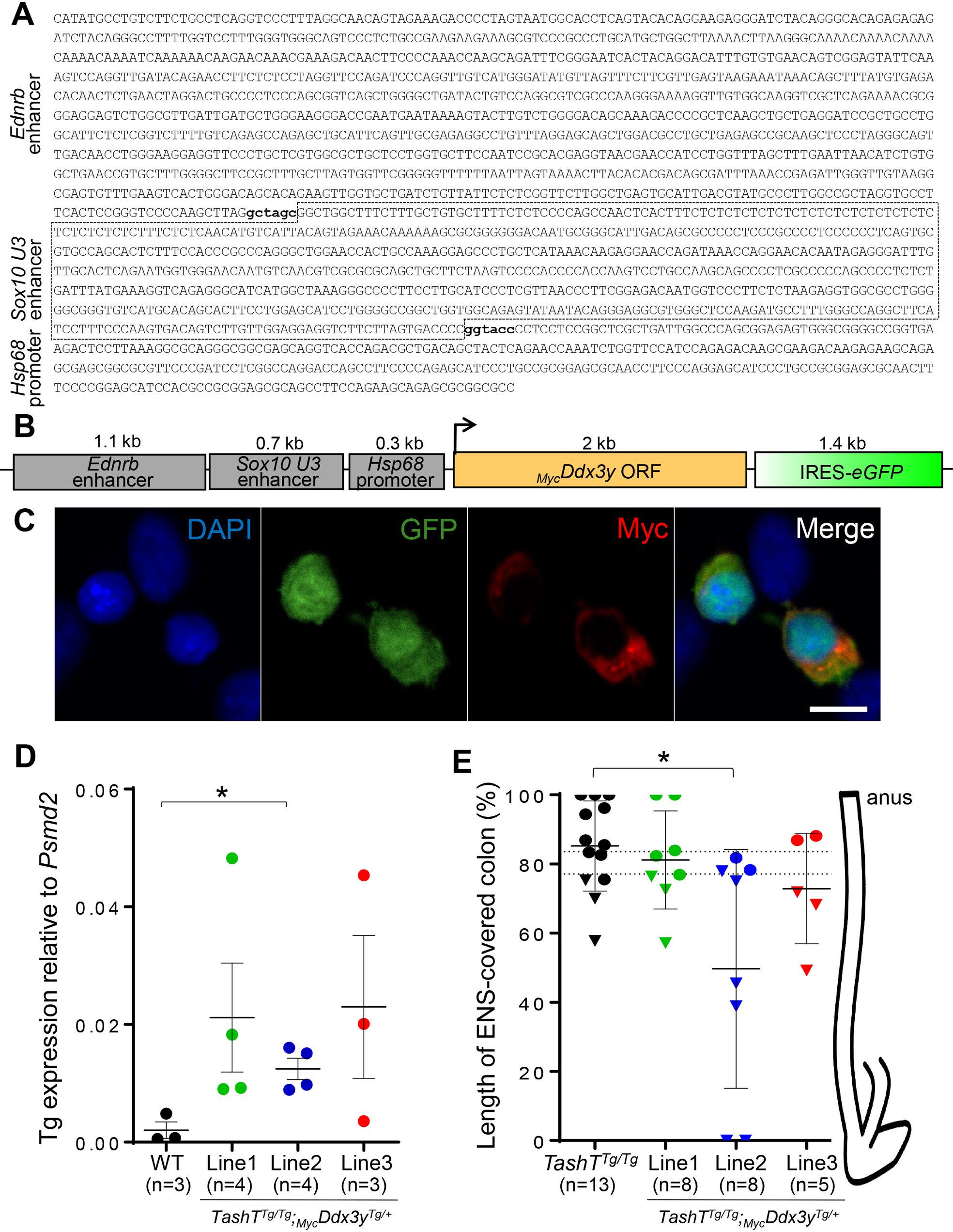

Supplement: S1 Fig — (A) Complete sequence of the Ednrb-Sox10-Hsp68 synthetic promoter. Restriction sites used for cloning are in lowercase letters. (B) Schematic representation of the MycDdx3y-IRES-eGFP transgenic construct, with regulatory sequences in grey. (C) Immunofluorescence labeling of Neuro2a cells transfected with the MycDdx3y-IRES-eGFP transgenic construct and showing co-expression of MycDdx3y (red) and GFP (green) proteins. Scale bar, 10μm. (D) RT-qPCR analysis of MycDdx3y transgene expression in e12.5 TashT Tg/+;MycDdx3yTg/+ ENCCs recovered by FACS. (E) Detailed quantitative analysis of the length of ENS-covered colon (in % of total colon length) in P18-P50 TashTTg/Tg mice bearing or not a transgenic allele from each of the three MycDdx3y transgenic lines. Every MycDdx3y transgenic line is identified using the same color code as in Fig 1E and 1F. The dashed lines show the previously described threshold level interval above which megacolon is less likely to occur in FVB/N mice [29]. (* P≤0.05; ** P≤0.01; Student’s t-test). (TIF) [file pgen.1009008.s001.tif]

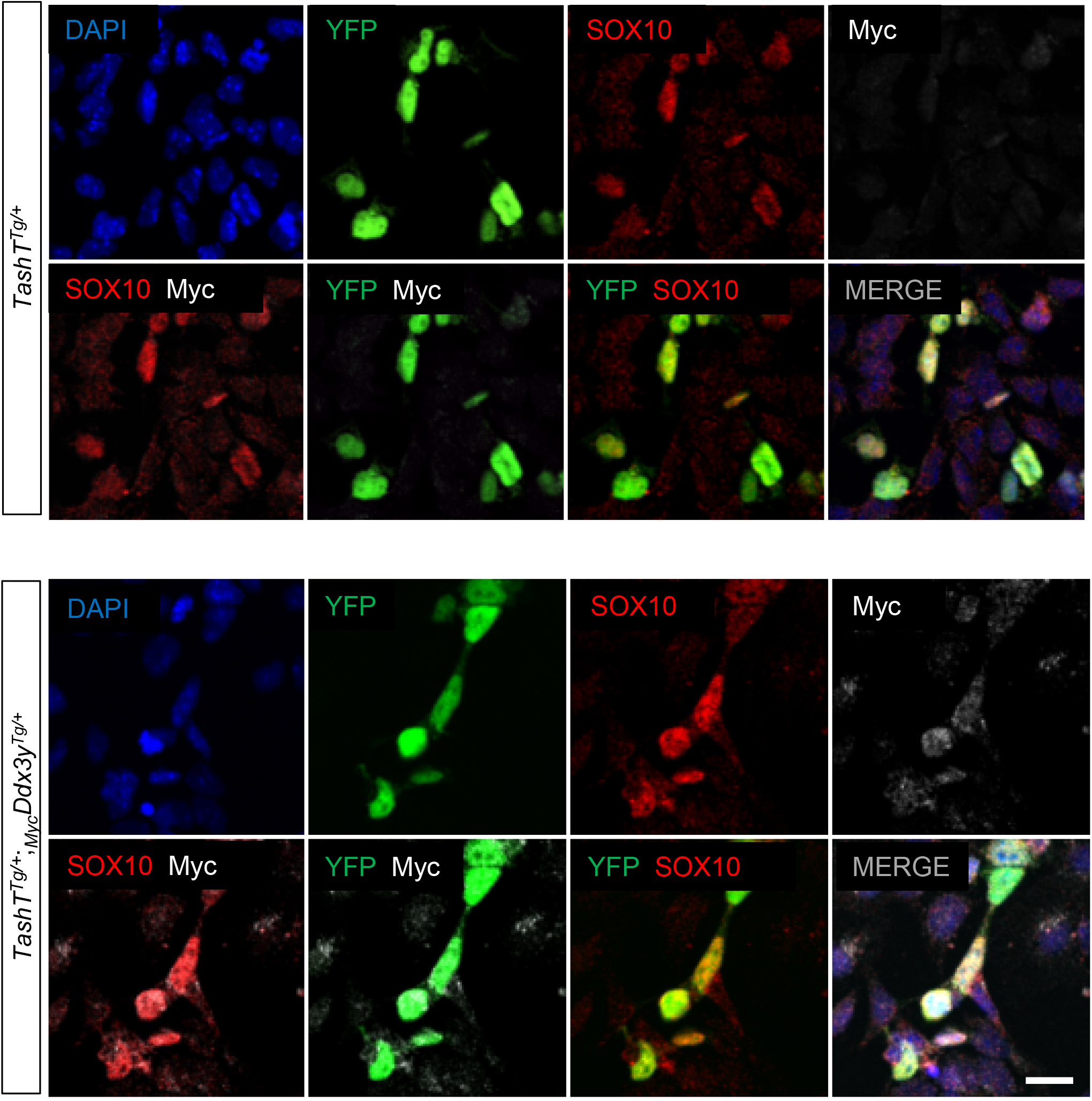

Supplement: S2 Fig — Representative confocal images of e12.5 midgut cells dissociated from TashTTg/+ control and TashTTg/+:MycDdx3yTg/+ (line 3) embryos, and immunolabeled for SOX10 (red) and Myc tag (grey). ENCCs are endogenously labeled with YFP owing to the pSRYp[1.6kb]-YFP transgene in the TashT line. Scale bar, 20 μm. (TIF) [file pgen.1009008.s002.tif]

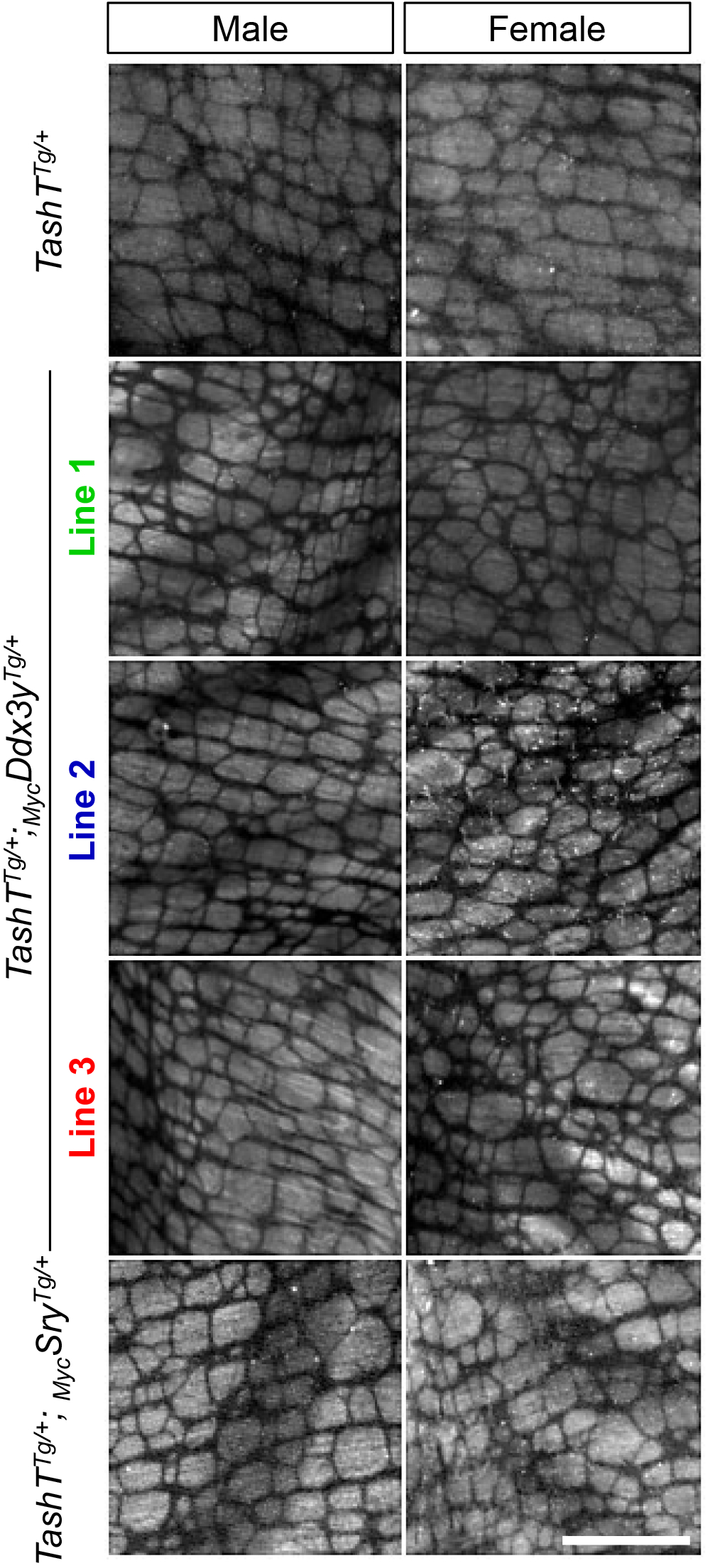

Supplement: S3 Fig — Representative images of proximal colon tissues collected from P18-50 TashTTg/+ mice bearing or not MycDdx3y or MycSry transgenic constructs and stained for AChE activity. Scale bar, 1000 μm. (TIF) [file pgen.1009008.s003.tif]

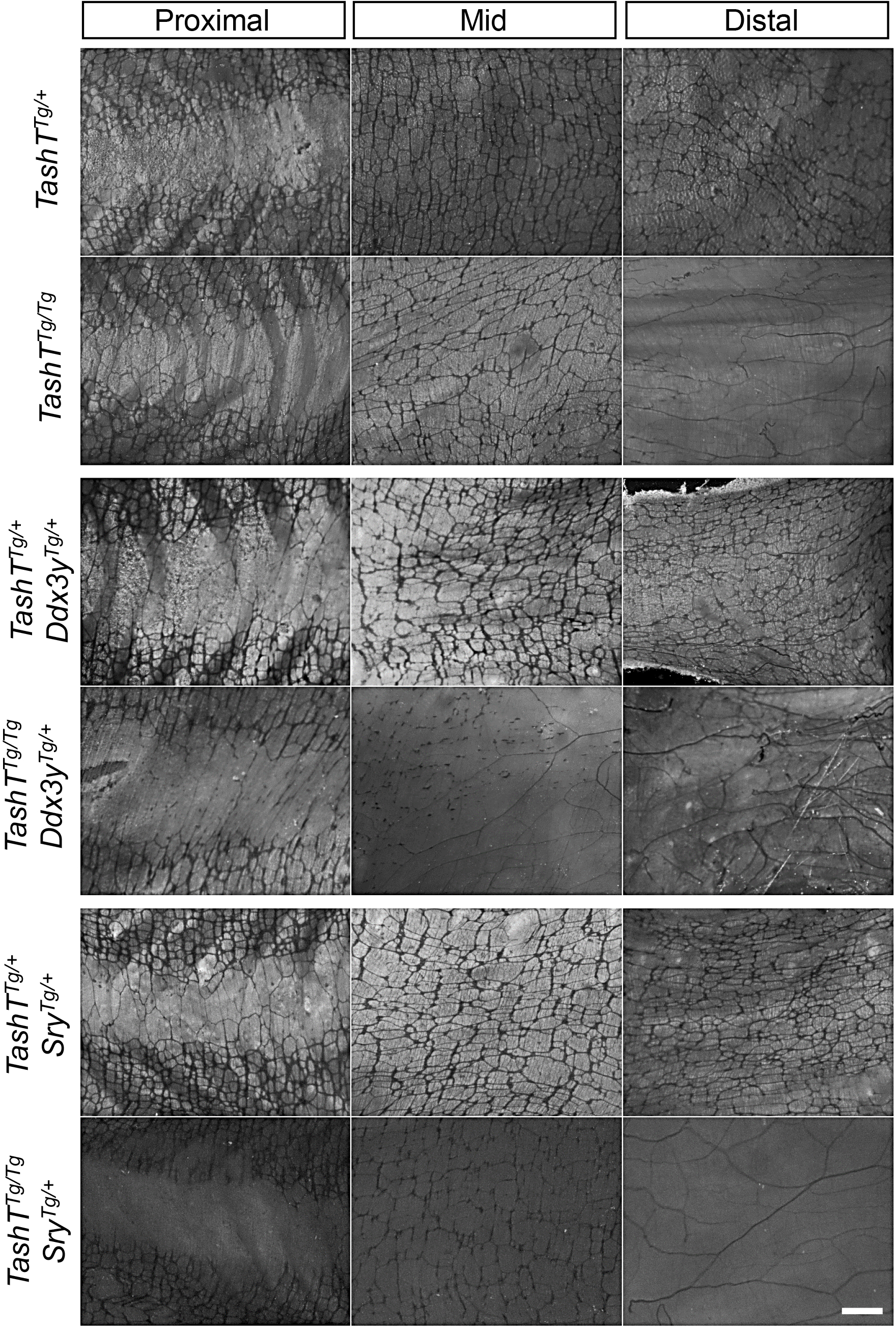

Supplement: S4 Fig — Representative images of P18-50 colon tissues collected from male mice and stained for AChE activity. Corresponding quantitative analyses are shown in Fig 1E and 1G. Scale bar, 1000 μm. (TIF) [file pgen.1009008.s004.tif]

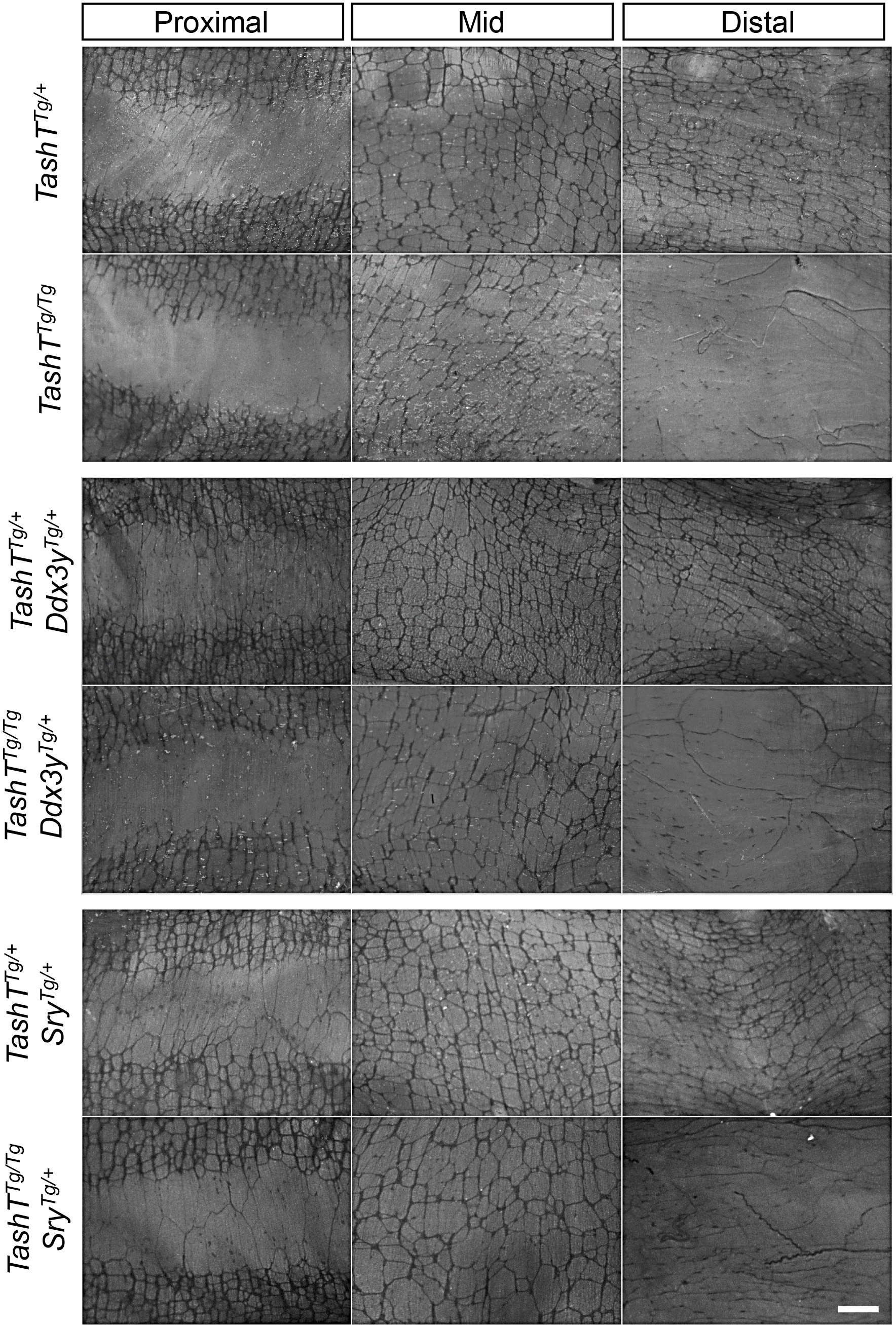

Supplement: S5 Fig — Representative images of P18-50 colon tissues collected from female mice and stained for AChE activity. Corresponding quantitative analyses are shown in Fig 1F and 1H. Scale bar, 1000 μm. (TIF) [file pgen.1009008.s005.tif]

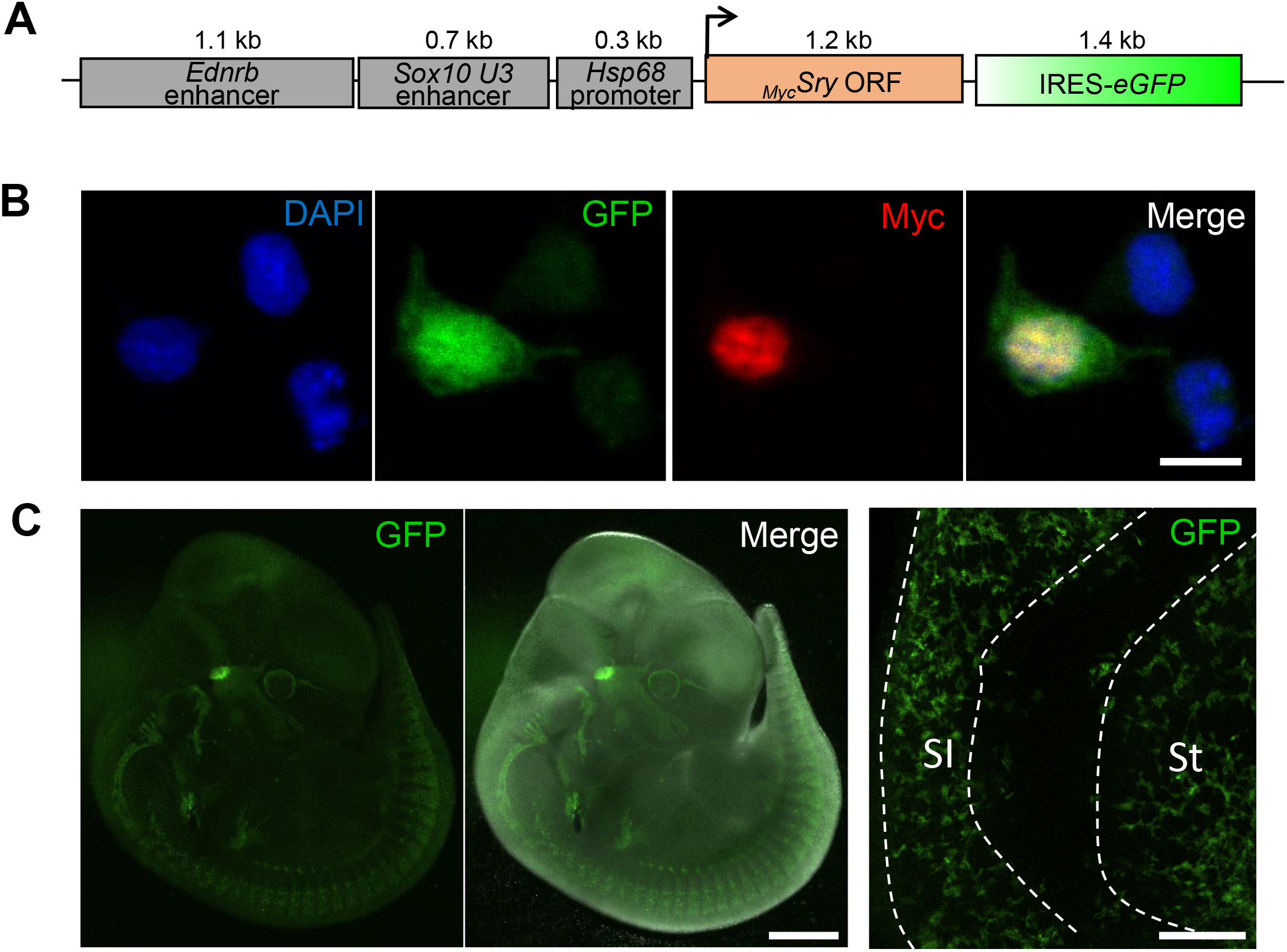

Supplement: S6 Fig — (A) Schematic representation of the MycSry-IRES-eGFP transgenic construct, with regulatory sequences in grey. (B) Immunofluorescence labeling of Neuro2a cells transfected with the MycSry-IRES-eGFP transgenic construct and showing co-expression of MycSRY (red) and GFP (green) proteins. Scale bar, 10μm. (C) Representative image of GFP-labeled neural crest-derived cells in e11.5 MycSry-IRES-eGFP transgenic embryos (n = 12). Left panels show GFP labeling of cranial nerves and dorsal root ganglia. Right panel is a high magnification view of the gut, to show GFP labeling of ENCCs in the prospective stomach (St) and small intestine (SI). Scale bar, 1000 μm (left panel); 200 μm (Right panel). (TIF) [file pgen.1009008.s006.tif]

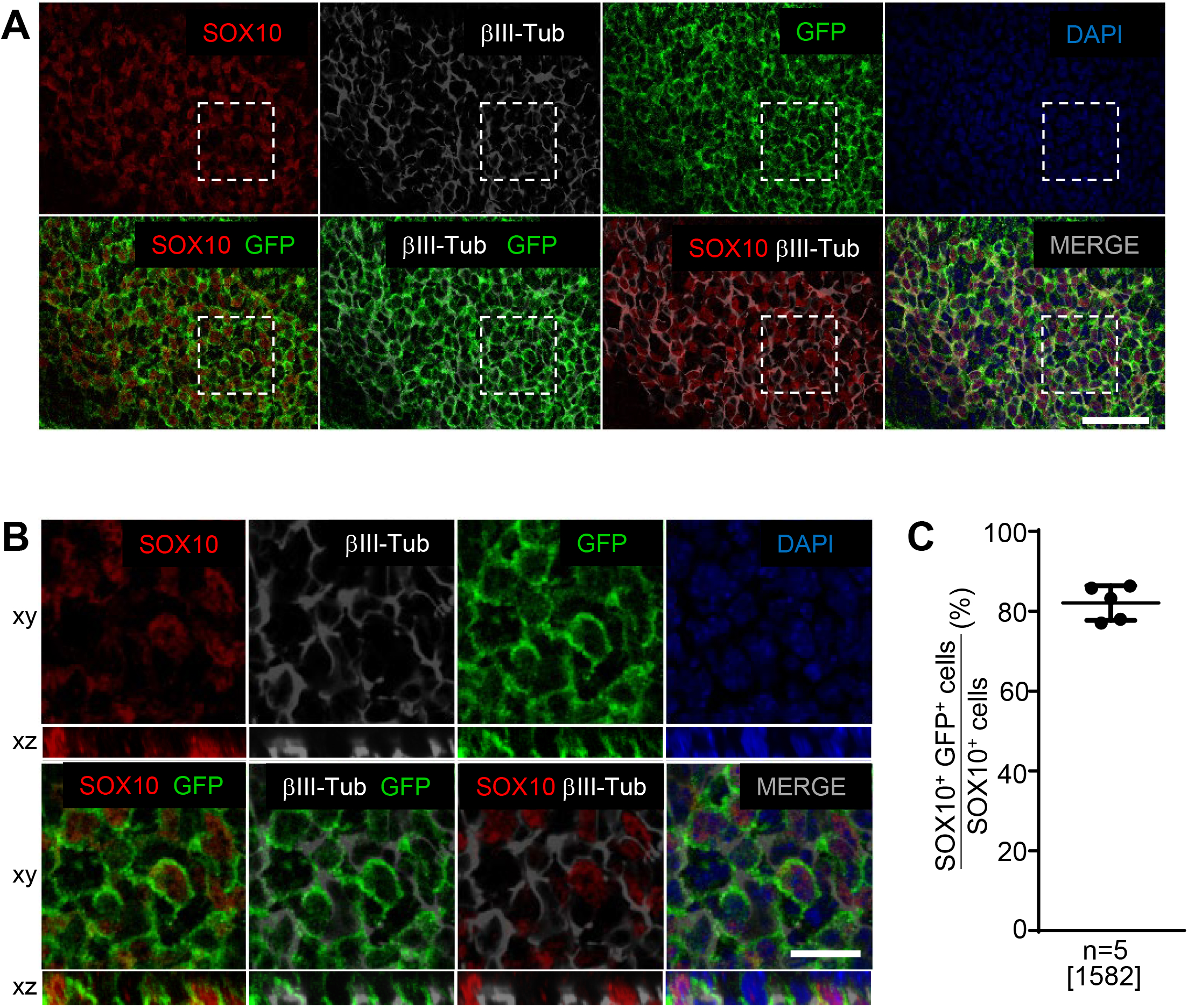

Supplement: S7 Fig — (A-B) Representative confocal images of e12.5 midguts collected from MycSryTg/+ embryos and immunolabelled for SOX10 (red), βIII-Tubulin (grey), GFP (green) and DAPI (blue). High magnification views shown in B are delineated by dashed square in A. As evidenced on both x-y and x-z planes, GFP signal is mainly present in SOX10+ cells. (C) Quantification of the number of SOX10+ GFP+ double positive cells on the total number of SOX10+ cells. The total number of counted cells is indicated in brackets. Scale bar, 50 μm (A) and 20μM (B). (TIF) [file pgen.1009008.s007.tif]

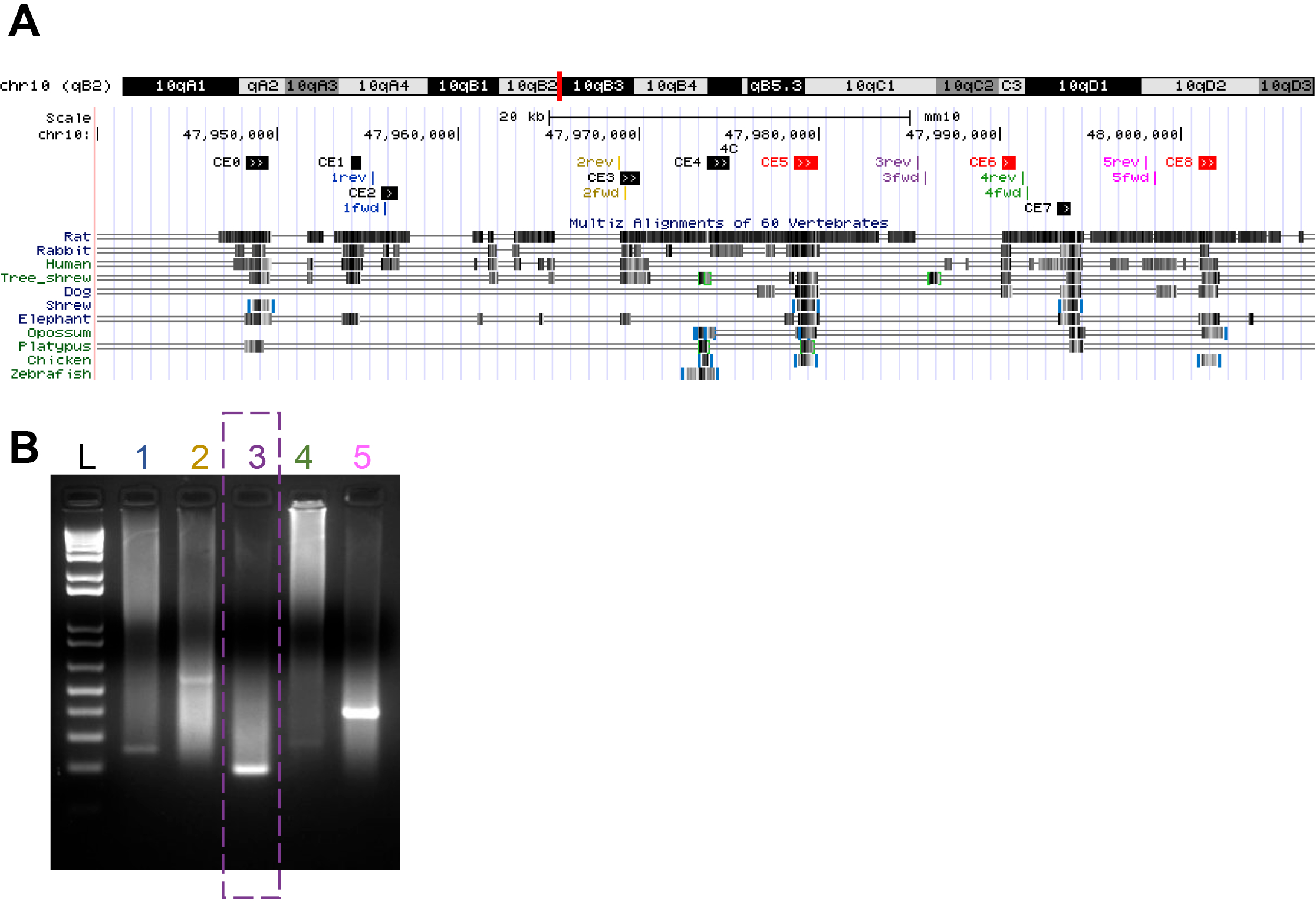

Supplement: S8 Fig — (A) Genomic representation of the Hace1-Grik2 intergenic region on chromosome 10, using the UCSC genome browser (genome.ucsc.edu) and its vertebrate Multiz Alignment & Conservation tract. Conserved elements (CE) are indicated by either black (neutral activity) or red (silencer activity) boxes, based on transcriptional activity in luciferase assays previously performed in Neuro2a and P19 cell lines [29]. Position of 4C-seq primer pairs is illustrated in blue (1), yellow (2), purple (3), green (4), and pink (5). (B) A test 4C-library was prepared using Neuro2a cells and used to evaluate efficiency of each primer pairs. The third pair (purple) was selected based on PCR profile and proximity to silencer elements. (TIF) [file pgen.1009008.s008.tif]

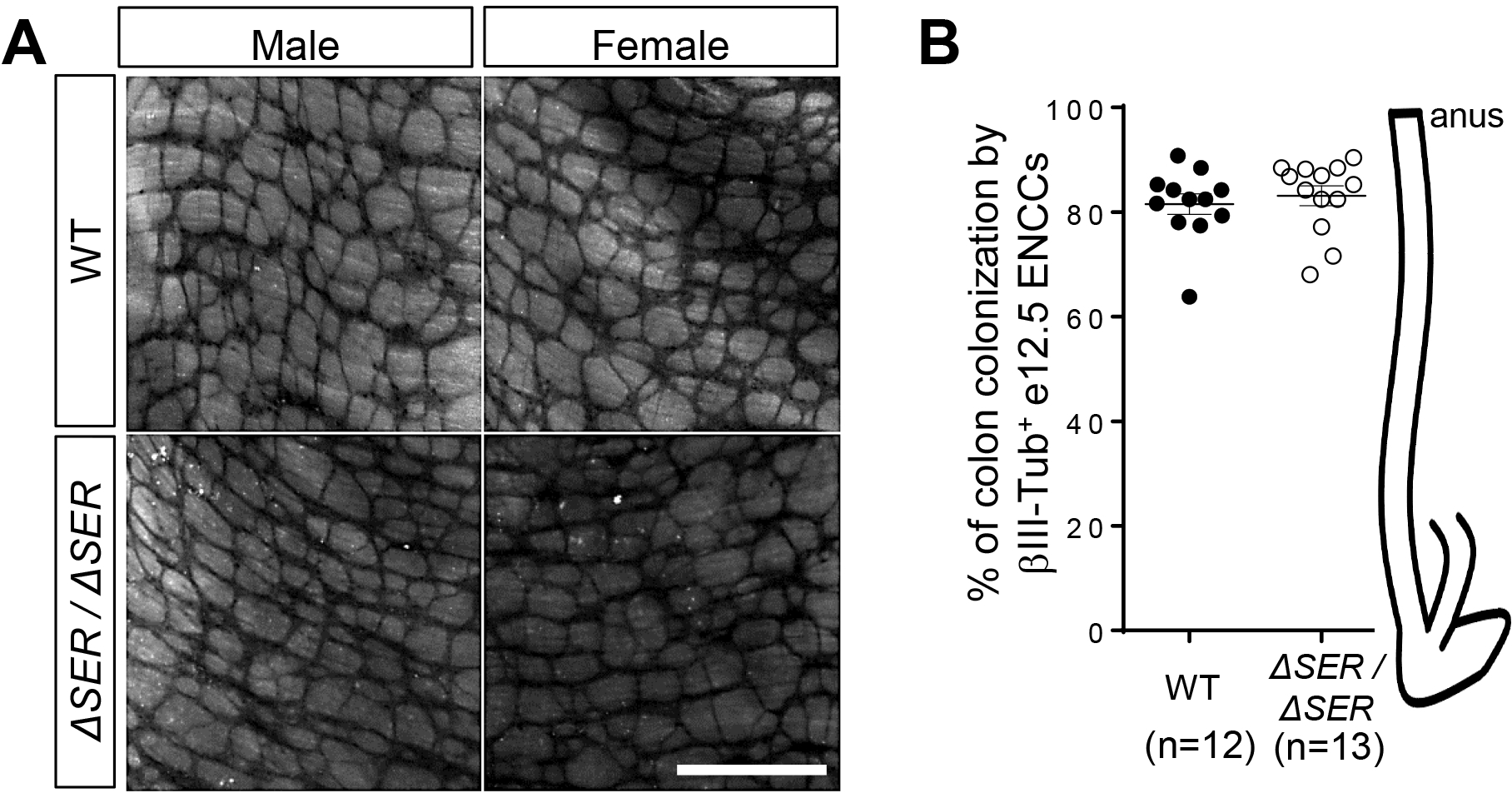

Supplement: S9 Fig — (A) Representative images of P18-50 proximal colon tissues collected from wild-type (WT; FVB/N control) and Hace1-Grik2ΔSER / ΔSER (ΔSER / ΔSER) mice, and stained for AChE activity. Lower magnification images are displayed in Fig 3C. (B) Quantitative analysis of the extent of colon colonization by βIII-Tubulin+ ENCCs (in % of total colon length) in WT and Hace1-Grik2ΔSER/ΔSER e12.5 embryos, showing normal colonization in mutant animals. Representative images are shown is Fig 3D. Scale bar, 1000 μm. (TIF) [file pgen.1009008.s009.tif]

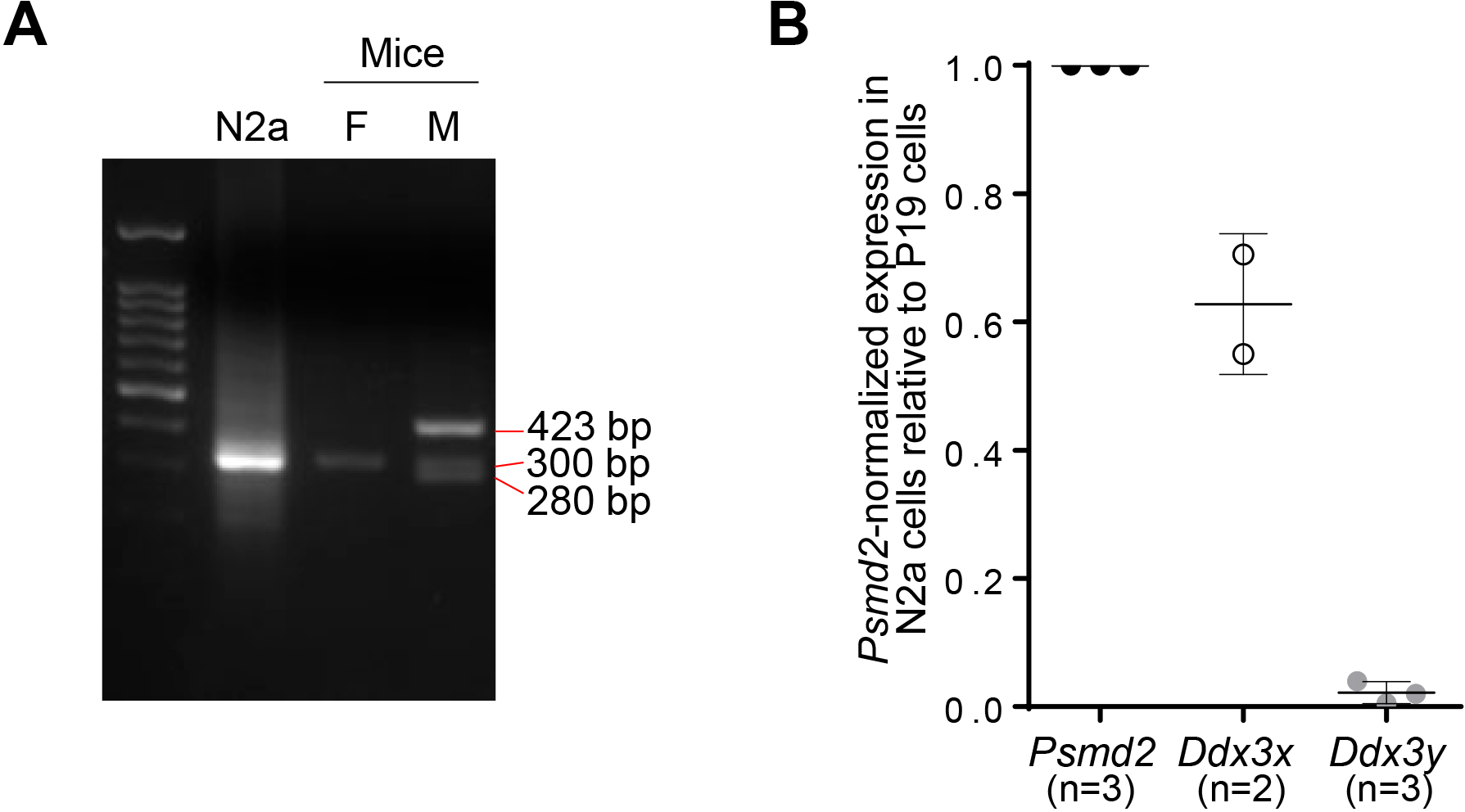

Supplement: S10 Fig — (A) PCR-based sexing of Neuro2a cells showing the presence of the X-linked gene SmcX (300 pb) but the absence of Y-linked genes Zfy (420 pb) and SmcY (280 pb). Genomic DNA from female (F) or male (M) mice was used as controls. (B) Relative expression of Psmd2 (autosomal gene), Ddx3x (X-linked gene) and Ddx3y (Y-linked gene) in Neuro2a compared to a known male mouse cell line (P19). Normalization to Psmd2 expression reveal the absence of Ddx3y expression. (TIF) [file pgen.1009008.s010.tif]
