# Supplementary material for: Male-biased aganglionic megacolon in the TashT mouse model of Hirschsprung disease involves upregulation of p53 protein activity and Ddx3y gene expression
Source: PLoS Genet. 2020 Sep 8;16(9):e1009008. doi: 10.1371/journal.pgen.1009008 (PMC7500598; doi:10.1371/journal.pgen.1009008)
Supplement: S1 Table — Full sequence of all oligonucleotides used in this study is shown in the 5’ to 3’ orientation. The purpose for which oligonucleotides were used is also mentioned. (PDF) [file pgen.1009008.s011.pdf]

S1 Table. List of all primers used in this study

**qPCR**	
**Eif2s3y_Fwd**	GAA TGG TTG GGC AGG TCC TT
**Eif2s3y_Rev**	GCC TCT TCT TAT CTG GCC CC
**Ddx3y_Fwd**	GTT GAC TGG TGG GGC AAT TG
**Ddx3y_Rev**	CCA GGA TAA GTT CAT TCA CCA TC
**Psmd2_Fwd**	CCC AGA CTA TGT CCT CGT CG
**Psmd2_Rev**	CCG TGT GAA GTT AGC TGC TG
**Ddx3x_Fwd**	AGT CGG TAT ATA AGG TCG GTG
**Ddx3x_Rev**	TGC TGT ACT TCC TCC ACT CTG
**Trp53_Fwd**	CCA TCA TCA CAC TGG AAG ACT CC
**Trp53_Rev**	TCC AGA CTC CTC TGT AGC ATG G
**IRES_Tg_Fwd**	TTG AAT GTC GTG AAG GAA GCA G
**eGFP_Tg_Rev**	GCA GAT GAA CTT CAG GGT CAG
**Cloning**	
**Ddx3y_cDNA_Fwd**	GTC GAC ATG AGT CAA GTG GCA GCG GA
**Ddx3y_cDNA_Rev**	AGA TCT TCA ATT GCC CCA CCA GTC AAC
**px330_CE05_gRNA_F1**	CACC G AAG GTG CAC GTC AGA ACT TC
**px330_CE05_gRNA_R1**	AAAC GAA GTT CTG ACG TGC ACC TT C
**px330_CE08_gRNA_F2**	CACC G CCA TAT GGC AAA TAT CAT CT
**px330_CE08_gRNA_R2**	AAAC AGA TGA TAT TTG CCA TAT GG C
**CRISPR/Cas9 targeting**	
**Cas9_Invitro_Fwd**	TAA TAC GAC TCA CTA TAG GGA GAA TGG ACT ATA AGG ACC ACG AC
**Cas9_Invitro_Rev**	GCG AGC TCT AGG AAT TCT TAC
**sgRNA_CE05_Invitro_Fwd**	TTA ATACGA CTC ACT ATA G AAG GTG CAC GTC AGA ACT TC
**sgRNA_CE08_Invitro_Fwd**	TTA ATACGA CTC ACT ATA G CCA TAT GGC AAA TAT CAT CT
**sgRNA_px330_Invitro_Rev**	AAA AGC ACC GAC TCG GTG CC
**Genotyping**	
**CRISPR_Hace-Grik2 SER_Fwd**	CAC TGT AAT CAC TTA ATG TTT GGT AAA CC
**CRISPR_ Hace-Grik2 SER _Rev1**	CTT CCC AGG AAA TTT TAA TGT AAC TGT TG
**CRISPR_ Hace-Grik2 SER _Rev2**	GGT ACC GGG GGT AAA CAT TTG ATC AGC ATC
**$_{Myc}$Ddx3y _Fwd**	ATC TAA AGG GGT CTG TGA TAA GG
**$_{Myc}$Ddx3y _Rev**	GAG AAT GGG TAG TAG AAA TGC AG
**$_{Myc}$Sry_Fwd**	TTT ATG GTG TGG TCC CGT GG
**$_{Myc}$Sry_Rev**	GAT GTC AGC TGT TAG TAA GTA GG
**Smcxy Forward**	TGA AGC TTT TGG CTT TGA G
**Smcxy Reverse**	CCA CTG CCA AAT TCT TTG G
**Zfy Forward**	GAC CAG ATT GTT GTG GAA GTA CAA G
**Zfy Reverse**	CCA GTG TGT CTG AAG TGT CAG CTG
**4C-seq**	
**4C_Pair 1_Fwd**	TAG GCT ACA GGT ATG TTT TTA GC
**4C_Pair 1_Rev**	ATC CCA GGT GTT GCC AAC AG
**4C_Pair 2_Fwd**	TGA AGG AGT TCT CAG TCA TTA AG
**4C_Pair 2_Rev**	CAA GTG ACT AGA AGG CAG ACC

**4C_Pair 3_Fwd**	CAT AGC CCT AGT TGT CCT GG
**4C_Pair 3_Rev**	GGA GGA ACC TTG ACC TGA TG
**4C_Pair 4_Fwd**	AA TTA GGT CTC TCT CCA TGC TG
**4C_Pair 4_Rev**	TAT CTA GGG AAT GAG CCC AAA C
**4C_Pair 5_Fwd**	CAG ACA CTA GAG TTT TCT GTG C
**4C_Pair 5_Rev**	CGC ATT GTA AAA CAC AAT TTA CTG G
**3C-PCR**	
**Dusp26 locus**	CCA GCA CCA CCC AAT TAT GTT AAA GGC TAC
**Hace1-Grik2 SER locus**	ACC CAA ATG AAG ACA GAA TGA GAA GTC ACC AGG AG
**ChIP-PCR**	
**pDdx3y_Fwd**	TAC CTT GGG TTG TGG AAT TGT G
**pDdx3y_Rev**	ACA AAG TGA AAA GGG TTG AGG C
**pDdx3x_Fwd**	TTG AAG CTG AGT CTT GGG GG
**pDdx3x_Rev**	AAT GTG TGA CTG GAC ACA ACC
**pMdm2_ Fwd**	AAA CGT CTT TCG GCA ATA GCT C
**pMdm2_ Rev**	GGT CCA GGA GGT GAC AGG T
