# Supplementary material for: Male-biased aganglionic megacolon in the TashT mouse model of Hirschsprung disease involves upregulation of p53 protein activity and Ddx3y gene expression
Source: PLoS Genet. 2020 Sep 8;16(9):e1009008. doi: 10.1371/journal.pgen.1009008 (PMC7500598; doi:10.1371/journal.pgen.1009008)
Supplement: Attachment — Submitted filename: Response to reviewers.docx [file pgen.1009008.s017.docx]

We sincerely thank the editors and reviewers for their careful review of our manuscript. As detailed below, we did our best to address all concerns raised by the reviewers, hoping that this will now make our manuscript suitable for publication.

**EDITORS**

1 - *Regarding the expression analyses that led to selection of Ddx3y there needs to be normalization of mutants to sex specific controls and greater explanation provided regarding the observed over expression of mesenchymal markers.*

**Response** : Although our strategy to compare our novel sex-stratified (*[Male]TashT*-*vs*-*[Female]Tash*) dataset to our previous unsexed *TashT-vs-Control* dataset might be seen as underpowered, it did lead to the identification of *Ddx3y*. Specific upregulation of *Ddx3y* (*vs* neighbour genes) in male *TashT* ENCCs was then confirmed by direct comparison with male control ENCCs (Fig.1C), and *in vivo* data (Figs.1E and Fig.5) are supportive of a functional role in the *TashT* male sex bias. We would have repeated our *TashT-vs-Control* analysis in a sex-stratified manner only if no dysregulated gene could have been identified, but this was not the case. As we are all asked to reduce the number of animals used in research, we felt it unnecessary and unethical to use more mice than needed to answer our question. In our opinion, there is no reason to believe that *Ddx3y* would not have stood out as primary candidate from perfectly sex-matched comparisons.

The question of mesenchymal markers with regards to specificity of ENCC labeling in *TashT* embryos has already been addressed in our published 2015 article (Bergeron et al., PLOS Genet 2015). The *pSRYp[1.6kb]-YFP* construct is expressed in mesenchymal cells, but only in the cecum and proximal colon (see Fig.2A of 2015 paper). In the regions we used for RNAseq (i.e., stomach and small intestine; as indicated in both Results and Materials/Methods sections of current manuscript), we have previously shown extensive colocalization of YFP with neuronal (βIII-Tubulin) and enteric progenitor/glial (SOX10) markers, without any expression in the mesenchyme (please see S1B Fig. of 2015 paper). The expression of mesenchymal/smooth muscle markers in *TashT* ENCCs is thus part of their molecular signature. As suggested by Rev.3, the presence of such markers in *TashT* ENCCs might be explained by abnormal cell fate switching toward neural crest-derived smooth muscle cells that are notably present around large blood vessels. With all due respect for the concerns raised, we don’t think that further commenting our previously published results for the *TashT* line would add anything to the current study focused on the *TashT* male bias.

2 - *Regarding the EdnrB/Sox10 enhancer construct, greater detail about this tool (including final sequence and expression relative to known ENS markers) needs to be provided to instill confidence in it's validity.*

**Response**: As requested, we added the complete annotated sequence of the *Ednrb-Sox10U3-Hsp68* synthetic promoter in S1A Fig. and a marker analysis in S7 Fig. This synthetic promoter is mainly active in SOX10^+^ cells, driving GFP expression in about 82% of them in e12.5 midgut (S7C Fig.).

3 - *Regarding the MycDdx3y-IRES-eGFP transgene additional effort should be made to show that Ddx3y protein is made from this construct, that the construct does not alter the patterning or composition of the ENS in transgenic mice, and the detail of which transgenic line (1, 2, or 3) were used to generate the data in subsequent figures within the manuscript should be included in the body of the text.*

**Response**: As requested, we put more effort into demonstrating expression of DDX3Y protein in transgenic tissues. As shown for line #3 in S2 Fig., we were finally able to make anti-Myc immunofluorescence working in freshly dissociated e12.5 midgut cells from lines 1 and 3 (we were unfortunately unable to obtain embryos from line #2 before the COVID19-related closure of our institute). In addition, we now show representative images of AChE staining showing that ENS patterning appears overtly normal in proximal colon, regardless of transgenic line or sex (S3 Fig.). Where relevant, we now also specifically mention which transgenic line was used to generate data.

**REVIEWER 1**

1 - *The study presents a combined Ednr/Sox10 enhancer construct to drive expression in the developing ENS. As this construct is new and previously uncharacterized, please provide data showing its efficiency and specificity in the developing gut (as well comment on the expression elsewhere in the embryo). An attempt is made for the mycSRY-IRES-eGFP construct, but a correlation to ENCCs at a cellular level is needed. Please provide percentage of Sox10 cells expressing the transgene, and the level of specificity within the gut. A validated ENCC enhancer-construct for expression in mouse ENS will be appreciated in the field at large.*

**Response**: Thank you for the suggestion and your interest in this tool. As indicated in our response to Editors’ comment #2, we added the complete annotated sequence of the *Ednrb-Sox10U3-Hsp68* synthetic promoter in S1A Fig. and a marker analysis in S7 Fig. In the ENS, this synthetic promoter is mainly active in SOX10^+^ cells, driving GFP expression in about 82% of them in e12.5 midgut (S7C Fig.). As commented in the Results section (line 231), this promoter also drives gene expression in other parts of the peripheral nervous system.

2 - *Introduction (row 71-73): The description of HSCR as loss of ganglia from distal colon is simplified. Approximately 20% of HSCR patients display long-segment or total colonic aganglionosis. A small adjustment of this sentence is required to account for these cases.*

**Response**: As suggested, this sentence has been adjusted to take into account all possible cases. Related information is also provided at lines 91-94.

**REVIEWER 2**

1 - *The finding that DDX3Y protein was only detected in 50% of male HSCR patients is somewhat problematic. It is curious that methods, other than immunofluorescence were not used to try to detect this protein. What is the percentage of expression of DDX3Y in different lines of mice? This result needs a bit more attention in the discussion.*

**Response**: Human tissue samples we have access to are already fixed and therefore not suitable for western blotting. Moreover, immunofluorescence offers the major advantage of seeing which cell types are expressing the protein of interest. We do not think that detection of DDX3Y protein in enteric neurons of 50% of male patients is problematic. DDX3Y does not have to be overexpressed in every male patient to contribute to the overall male bias. Moreover, as indicated in the Results section, it is important to bear in mind that DDX3Y is expected to have a pathogenic role before birth (i.e., during ENS formation). Because HSCR is diagnosed only after birth, it is impossible to analyze HSCR tissues at most relevant developmental stages. Nonetheless, we reasoned that at least some residual expression might still be detectable in the ENS-containing regions of some male patients a few weeks/months after birth. Our discovery that half of male patients do express DDX3Y protein in the ENS after birth is therefore remarkable. Yet, this result should be interpreted with caution since the percentage of male patients expressing DDX3Y after birth does not necessarily match the proportion before birth (could be more or less than that). As suggested by this reviewer, we further commented this result in both Results and Discussion.

2 - *Although the studies designed to assess potential differences between Ddx3x and Ddx3y in their regulation by P53 are important, the use of the cell lines chosen makes interpretation of these data difficult. Gene regulation in each of these lines is not “normal.” Each of these lines has multiple issues related to gene expression especially of neural crest related genes. It would be interesting to repeat this experiment in sorted ENCCs which can be efficiently grown in culture and which are amenable to knock-down. Alternatively, ENS-derived ENCCs efficiently develop embryoid bodies in culture.*

**Response**: Thank you for the suggestion. To be as close as possible to *in vivo* conditions, we chose to address this concern using ENCCs recovered by FACS from *TashT^Tg/Tg^* embryos exposed or not to the p53 inhibitor pifithrin-α (Fig.5). This allowed us to not only confirm our findings with R1 and F9 cell lines regarding the preferred regulation of *Ddx3y* over *Ddx3x* (Fig.5C-D) but also to find a robust inverse correlation between *Ddx3y* expression levels and extent of ENCC colonization (Fig.5E).

3 - *Inasmuch as all male mice did not develop HSCR in response to altered expression of Ddx3y, it is an overstatement that this gene is responsible for the male bias of HSCR in all mice; please tone this down.*

**Response**: we toned it down as requested.

REVIEWER 3

1 - *The authors wrote the methods section that frequently required the reviewer to refer back to their earlier reports. Example: “Analyses of endogenous YFP fluorescence was performed as previously described.”. This required the reviewer to refer back to their earlier data and understand how the techniques that were central and crucial to understanding the experiments in this manuscript were performed.*

**Response**: we carefully revised our manuscript to make sure sufficient information is provided about how experiments were performed, either in the Results or in the Materials/Methods section.

2 - *On referring back to the earlier reports, the reviewer found that the YFP fluorescence in the TashT line in colon but not in small intestine is also expressed in the mesenchymal cells. Indeed the data presented in this manuscript shows that in the TashT vs Control gene expression differences from the YFP/GFP+ ENCC from small intestine and stomach (Dataset S2), the TashT transgenic animals have an overexpression of mesenchymal markers (Acta2, Actg2, Myh11) compared to controls. They also include genes such as Lumican and Col6a4 that are important for the normal migration of ENCC. Now this either means that the fidelity of the sort is compromised and that the TashT dataset contains contaminating genes from YFP- non-ENCC or that TashT causes some kind of fate-transition of these neural crest-derived cells towards a mesenchymal fate. In any case, this dataset is the basis of identification of the gene expression differences between normal and transgenic animals and these important issues have not been addressed adequately. The reviewer is careful not to critique the past reports and is confining himself/herself to this report where this data has been presented and is central to the identification of Ddx3y in the next screen.*

**Response**: As mentioned in our response to Editors’ comment #1, the question of mesenchymal markers *vs* specificity of ENCC labeling in *TashT* embryos has already been carefully addressed in our previous study (Bergeron et al., PLOS Genet 2015). Dataset S2 has been generated in the context of this previous study, and was included in the current manuscript for convenience purposes only. This Reviewer is right in noting that the *pSRYp[1.6kb]-YFP* construct in the *TashT* line is expressed in mesenchymal cells of cecum and proximal colon only (see Fig.2A of 2015 paper). For this reason, we always perform our gene expression analyses using the stomach and small intestine (as indicated in both the Results and Materials/Methods sections), tissues in which we previously demonstrated extensive colocalization of YFP with ENCC markers without any expression in the mesenchyme (please see S1B Fig. of 2015 paper). As suggested by this reviewer, the expression of the indicated mesenchymal/smooth muscle markers by *TashT* ENCCs might well be explained by abnormal cell fate switching toward neural crest-derived smooth muscle cells (which are notably present around large blood vessels). In any case, these genes, like the vast majority of the other dysregulated genes in *TashT* ENCCs, were eliminated during the comparative analysis that led to the identification of *Ddx3y*.

3 - *The intriguing part of the datasets presented here and that have not been commented on is that the TashT animals have an overall increase in expression of Gdnf but an underexpression of Ret and Gfra1 as compared to controls.*

**Response**: This result, as well as the similar *Edn3* upregulation / *Ednrb* downregulation, is indeed intriguing. This is why it has been abundantly commented in the Discussion of our 2015 article (please see pages 14-15 in Bergeron et al., PLOS Genet 2015). We do not think that further discussing this is relevant in the context of the current study.

4 - *In addition, the sex-biased TashT gene expression analyses has not been normalized each sex of mutant mice to their own controls. An ideal and correct experiment would be to perform TashT males vs Control males and TashT females vs Control females and then compare the significantly different genes between these two datasets. In the current experimental design and analyses, there would be an expected domination of X and Y linked genes and the study becomes underpowered and important genes that are present on autosomes and are differentially regulated between males and females do not get tested. The authors are advised to perform these analyses so that the transgenic associated sex-differences can be baselined correctly.*

**Response**: As indicated in our response to Editors’ comment #1, even though our strategy to compare a sex-stratified *TashT* dataset to an unsexed *TashT-vs-Control* dataset might be seen as underpowered, it did lead to the identification of *Ddx3y*. Moreover, 25% (2/8) of genes showing sex biased dysregulation are located on autosomes (Fig.1B), meaning that at least a subset of such genes did get tested. It is also noteworthy that we took care to confirm the specific upregulation of *Ddx3y* (*vs* neighbour genes) in male *TashT* ENCCs by direct comparison with male control ENCCs (Fig.1C). In addition, we provide compelling *in vivo* evidence that *Ddx3y* overexpression can play a role in the *TashT* male sex bias (Figs.1E and Fig.5). Considering all our findings and the legitimate pressure to reduce the number of animals used in research, we did not find it necessary nor ethically justified to repeat our *TashT-vs-Control* analysis in a sex-stratified manner. We are convinced that if *Ddx3y* could be picked up using a one-side sex-stratified approach, it would also have been picked up using a two-side sex-stratified approach.

5 - *As a control for Ddx3y (Fig 1C), the authors chose Eif2s3y, a gene that is significantly upstream and is in the opposite direction. The authors do not provide any rationale for why this gene was chosen instead of the gene Uty that is immediately next to Ddx3y and is in the same direction.*

**Response**: *Uty* is now also included in this analysis (Fig.1C).

6 - *The authors used three transgenic lines of Ddx3y overexpression in the TashT background and a cumulative analyses shows a significant male biased aganglionosis and megacolon (Fig 1 E, F). However, Fig S1 show that there are major variations between the three lines. While all three lines show significant differences compared to WT (Fig S1C), only the second line shows any significant difference in aganglionosis compared to the TashT background. The reviewer then wonders why the other lines were included in the main data analyses or why the Lines 1 and 3 that show a significant overexpression of Ddx3y fail to show significant increase in the length of aganglionosis (Fig S1D). This combined with the data that shows that overexpression of Ddx3y in females had no effect makes the reviewer wonder whether Ddx3y is really that central to the male bias.*

**Response**: We think that our study is much more convincing with all three lines included. All three were kept in this study because they all showed a marked increase in the proportion of animals displaying megacolon. From 23% (3/13) of *TashT* males, megacolon incidence reached 37.5% (3/8) for line#1, 75% (6/8) for line #2, and 60% (3/5) for line #3 (S1E Fig.). We also believe that the association with extent of aganglionosis is best interpreted through the lens of the minimal length of intrinsic innervation (~80% of total colon length) necessary to avoid blockage in the FVB/N genetic background (see Fig.1E in Bergeron et al., PLOS Genet 2015). Although statistical significance in the average extent of aganglionosis was not reached for lines #1 and #3 (most likely because of small sample size), increased severity of aganglionosis was in each case sufficient to markedly increase the proportion of animals not reaching this minimal length of intrinsic innervation. The corresponding section in the Results has been completely re-written to better explain these results and their associated interpretation (lines 210-220).

7 - *The authors implicate sex-specific trans-activation of p53 is in the male-biased phenotype. However, the controls themselves show what seems to be a significant reduction in p53 expression (Fig 4F). This is very important but this is neither statistically tested by the authors nor the implications of this tested and discussed.*

**Response**: As now indicated in Fig.4F and accompanying text in the Results section, the noted male-biased decrease of p53 activity in control ENCCs is statistically significant. We fully agree that this sex-based difference is potentially meaningful, especially when considering our findings suggesting preferential regulation of *Ddx3y* in male cells. As now indicated in the Discussion, this might be indicative of a safeguard mechanism aimed at preventing *Ddx3y* expression to reach pathogenic levels in male ENCCs.

8 - *Fig 5 shows that the addition of a p53 inhibitor to the Ddx3y – TashT mice abrogates the male bias. However, the correct way of analyzing this would be to compare each drug-treatment to its own sex-specific control. Analyzing it in that manner shows that there is a large variation and that the drug treatment is either subclassifying the effect into two populations (where one population has an effect and other doesn’t) or that the drug dosage and timing isn’t optimized.*

**Response**: Thank you for the suggestion. As shown in revised Fig.5B, we increased *n* numbers for all groups and performed supplemental statistical comparisons. Adding more samples eliminated the apparent subclassification into two groups and strengthened our rescue data, which now more clearly show the male-specific impact of pifithrin-α treatment.

9 - *The authors should also present what the Ddx3y immunoreactivity in the aganglionic part of the murine and human gut look like.*

**Response**: As indicated in the Materials/Methods section, our human samples were all collected from the ENS-containing transition zone where we found DDX3Y immunoreactivity to be restricted to enteric neurons. This means that, even if we would have had access to aganglionic tissues, no expression would have been expected in such ENS-free region. It is also important to note that Sigma’s mouse monoclonal anti-DDX3Y antibody used for this work is specific for the human DDX3Y protein. As we experienced ourselves, this antibody does not work in mouse tissues. Moreover, to the best of our knowledge, there is no commercially available antibody for the specific detection of mouse DDX3Y protein (all existing ones are mentioned to cross-react with DDX3X).

10 - *DataS4: the nomenclature of Unique to and Common to is confusing and doesn’t describe whether genes in those datasets are specific to one or other groups.*

**Response**: We did not find a better way to classify all 15 categories of the Venn diagram displayed in Fig.4A. Each tab of the spreadsheet corresponds to one of these 15 categories, and therefore contains a list of genes that are exclusively present in a given category. To help matching Venn diagram categories and corresponding lists of genes, the number of genes per category is now indicated in every tab’s name.

11 - *Line 185: The authors should say “associated with” rather than “responsible”*

**Response**: we toned it down by saying “might contribute”
