# Supplementary material for: Male-biased aganglionic megacolon in the TashT mouse model of Hirschsprung disease involves upregulation of p53 protein activity and Ddx3y gene expression
Source: PLoS Genet. 2020 Sep 8;16(9):e1009008. doi: 10.1371/journal.pgen.1009008 (PMC7500598; doi:10.1371/journal.pgen.1009008)
Supplement: Attachment — Submitted filename: Response to reviewers_R2_FINAL.docx [file pgen.1009008.s018.docx]

We sincerely thank the editors and reviewers for the re-review of our manuscript. As detailed below, we carefully addressed all remaining concerns and hope that you will find our manuscript suitable for publication.

**EDITORS**

*1 - As currently presented, the manuscript implies that DDX3Y is the only sex-associated transcript that exhibits differential expression. If the manuscript is to retain this stance, comparing the TashT males vs Control males and TashT females vs Control females is an important aspect that is should be done and incorporated. Please take this aspect into consideration when revising the manuscript. If current circumstances prevent this analysis, one alternative is to restructure your presentation in the text so that this statement is toned down.*

**Response**: Although we disagree with the Reviewer-3’s comments that apparently underly this request, we added a sentence (lines 186-188) that we hope will be satisfactory: “Although a fully sex-stratified RNA-seq approach (*i.e.*, comparing *TashT^Tg/Tg^* males and females to control males and females, respectively) might potentially have identified additional genes,…”

*2- As previously commented upon in the initial reviews the manuscript infers that there is a causal link between the change in DDX3Y expression on p53 inhibition and the reduction/absence in aganglionosis in the TashT mouse. Definitive support for this statement could be generated by showing that there is no change to aganglionosis when p53 is inhibited in the TashT-DDX3Y over expression mouse. Alternatively, you could revise the text and the paper title to remove this over-interpretation of current information and indicate that your results suggest an interaction that could be investigated in the future.*

**Response**: We agree that the p53-Ddx3y link could be strengthened by the proposed experiment but chose the proposed option to revise both title and text in order to tone down our interpretation.

**REVIEWER 1**

*The revised version include the sequence of the EdnrB/Sox10 construct and quantification of the proportion of Sox10 cells where the construct is active. For transparency I only ask for two more details:*

*1) Please add the individual data-points in your bar-graph in Figure S7. Although not mentioned in the first review, it is highly advisable to include data-points in all graphs included in the manuscript.*

**Response**: data-points have been added to all relevant graphs.

*2) Please add a picture of a transverse cut through the intestine showing the expression of YFP/SOX10/BIIITub to indicate the specificity of expression within the ENS.*

**Response**: A virtual transverse section (x - z plane) has been added under each panel of S7B Fig.

**REVIEWER 2**

*This study provides strong evidence that the p53-DDX3Y regulatory axis is at least partially responsible for the male bias in Hirschsprung’s disease (HSCR). This is a very interesting and potentially important finding; the authors have identified a potential treatment target for subsets of patients with HSCR. This data are clearly presented and the studies are well controlled. The authors have done an excellent job of addressing all of the issues raised in the previous review. This manuscript will make a significant contribution to the ENS field in general and to those most interested in HSCR.*

**Response**: Thank you so much for these very positive comments.

**REVIEWER 3**

*The authors in this revised manuscript have addressed many of the comments put forth by the reviewer in the prior review to varying degrees of satisfaction. The reviewer will address the remaining issues in order.

1. The reviewer thanks the authors for significantly re-writing the manuscript to put forth their point of how the sex-stratified analyses of TashT mice was done and how the identification of the gene DDX3Y could have been made even with the strategy suggested by this reviewer. While the reviewer does not disagree that DDX3Y could have been identified in the other manner as well, the reviewer would humbly suggest that the suggested strategy would provide more and clearer understanding of the genetic factors altered in the TashT mice that make male mice more susceptible to aganglionosis. The reviewer would refer the authors to their Fig 4f, which now shows that the expression of p53 gene is significantly different in an analyses of (female control to female TashT) and (male control to male TashT). As a result, with the analyses the reviewer suggested, the authors would have easily picked up p53 as a gene that is expressed more than 2 fold differently between the TashT mice of the two sexes. While the current analyses is fine for identification of DDX3Y, it gives an erroneous impression that only this gene is significantly different and hence is the only key gene implicated in male bias in TashT aganglionosis. Such an analyses also might hold the key to explaining why DDX3Y overexpression only causes aganglionosis in the TashT background, which is the question posed by the authors in their Discussion.*

**Response**: As indicated in our response to Editors’ comment #1, we added a sentence about the possibility of having missed additional genes because of our non-fully sex-stratified approach (see lines 186-188). However, we totally disagree with the reviewer’s interpretation of our p53 data. Fig.4F does not refer to *Trp53* mRNA but to p53 protein level, which is widely used as proxy for stabilized/activated p53 protein (as now more clearly indicated where relevant in both text and figure). *Trp53* mRNA levels do not generally vary. The encoded p53 protein is instead well-known to be regulated at the post-translational level, and most especially via phosphorylation (and this is exactly why the p53 phosphatase-coding gene *Dusp26* caught our interest). In absence of cellular stress, unphosphorylated p53 protein is otherwise quickly degraded and replaced through steady *Trp53* mRNA transcription/translation, which ensures maintaining basal levels of p53 protein. In line with this mode of regulation, *Trp53* mRNA levels are not significantly affected in unsexed *TashT* vs control ENCCs (please see line# 11086 of S2 Dataset in Bergeron et al., *PLOS Genet* 2015; now also mentioned in current manuscript at lines 326-328). In marked contrast, *Ddx3y* already appeared dysregulated even in this unsexed analysis. For these reasons, it is highly unlikely that the *Trp53* mRNA would have been picked-up even with the approach proposed by the reviewer.

*2. The reviewer congratulates the authors on showing strong data on the effect of p53 inhibition on aganglionosis in the TashT mouse line. However, the reviewer feels that the causal axis of p53 and DDX3Y proposed by the authors is tenuous, since the authors did not check what would happen with p53 inhibition to aganglionosis when DDX3Y is overexpressed. The experiment to really nail the inference that "Male-biased aganglionic megacolon in the TashT mouse model of Hirschsprung disease involves upregulation of the p53-DDX3Y regulatory axis" (which is the title of the manuscript) would require the authors to show that there is no change to aganglionosis when p53 is inhibited in the TashT-DDX3Y overexpression mouse. This would provide clear and unequivocal evidence that there is a p53-DDX3Y regulatory axis that is key to the male bias of HSCR associated aganglionosis.*

**Response**: Thank you for the suggestion. As mentioned in our response to Editors’ comment #2, we agree that the link between p53 and Ddx3y could be strengthened using the proposed approach. However, we instead chose the option to revise both title and text in order to tone down our interpretation.

*3. As a minor point, the reviewer would advise the authors to streamline their manuscript to allow for a better and a more focused read.*

**Response**: We disagree with this point of view, like we believe both of the other reviewers do (based on their comments in the previous round of review):

Rev1: *“The manuscript is overall well-written and organized. All conclusions are sound and appropriately discussed.”*

Rev2: *“The transcriptomic approach to identification of differentially expressed genes in male vs female is excellent. It should be noted that this manuscript is very well written and the data beautifully presented.”*
